# Clinical performance of a hydrophobic acrylic toric intraocular lens with a double C-loop haptics in Japanese patients

**DOI:** 10.1007/s10384-025-01274-4

**Published:** 2025-09-29

**Authors:** Yosai Mori, Kazunori Miyata, Takashi Kojima, Kazuo Ichikawa, Yoshifumi Fujita, Takuya Shiba, Hiroko Bissen-Miyajima

**Affiliations:** 1 Miyata Eye Hospital, 6-3 Kurahara-cho, Miyakonojo, Miyazaki 885-0051 Japan; 2 Chukyo Eye Clinic, Nagoya, Aichi Japan; 3 Fujita Eye Clinic, Tokushima, Tokushima Japan; 4 Roppongi Shiba Eye Clinic, Minato-ku, Tokyo, Japan; 5 Department of Ophthalmology, Tokyo Dental College Suidobashi Hospital, Chiyada-ku, Tokyo, Japan

**Keywords:** Intraocular lens, Cataract, Astigmatism, Monofocal toric, Double C-loop

## Abstract

**Purpose:**

To evaluate the clinical performance of a hydrophobic acrylic toric intraocular lens (IOL) with double C-loop haptics in a Japanese population with cataracts.

**Study design:**

Prospective

**Methods:**

The PODEYE TORIC IOL (POD T 49P) was implanted in 58 eyes from 42 patients diagnosed with bilateral cataracts with corneal astigmatism. Thirty-one eyes received IOLs with cylinder powers of 1.50 to 6.00 D (group A), and 27 eyes received IOLs with 1.00 D cylinder (group B). Uncorrected (UDVA) and corrected (CDVA) distance visual acuities, and refractive cylinder were examined postoperatively. The primary endpoints, which were the non-inferiority of UDVA in group A and superiority of the refractive cylinder in group B, were examined and compared with relevant previous data.

**Results:**

In group A, the mean preoperative corneal astigmatism was 1.87±1.01 D and postoperative logMAR UDVA and CDVA were -0.023±0.110 and -0.102±0.079, respectively. There were 23 eyes (74.2%) with UDVA of 0.0 logMAR or better, while the refractive cylinder was - 0.39±0.39 D. In group B, the preoperative corneal astigmatism of 0.73±0.22 D and postoperative logMAR UDVA and CDVA were -0.074±0.091 and -0.096±0.075, respectively; 23 eyes (85.2%) obtained a UDVA of 0.0 logMAR or better. The postoperative refractive cylinder was -0.16±0.23D. The non-inferiority of UDVA in group A and the superiority of the refractive cylinder in group B were verified with previous data.

**Conclusion:**

The PODEYE TORIC IOL with 1.00–6.00 D cylinder powers and double C-loop haptics effectively corrected corneal astigmatism in Japanese patients after cataract surgery.

**Trial registration number:**

NCT04699266 (Clinicaltrials.gov)

## Introduction

In recent cataract surgery, corneal astigmatism has been corrected with the implantation of toric intraocular lenses (IOLs) by adding cylindrical power at the flatter meridian. In 110,468 cataract eyes in the United Kingdom, preoperative corneal astigmatism of 0.5 D or greater and 1.0 D or greater were found in 78% and 42%, respectively [1]. In Japan, an investigation of 12,428 cataract surgery eyes, shows a distribution of corneal astigmatism of 28.1% below 0.5 D, 35.6% above 0.5 D to 1.0 D, 7.4% above 1.0 D to 1.5 D, 7.4% above 1.5 D to 2.0 D, 3.8% above 2.0 D to 2.5 D, and 2.4% above 3.0 D [2]. Thus, the benefits and harm associated with the implantation of toric IOLs are recognised for corneal astigmatism of 1.0 D or higher [3]. Conversely, the efficacy of lower astigmatism has rarely been evaluated, as the influence would be limited and there would be no degradation in visual acuity with corneal astigmatism <0.5 D in young patients [4]. Recent findings on the influence of against-the-rule and oblique astigmatism [5], and posterior corneal astigmatism [6] show the importance of correction for 1.0 D or less; however, the performance of toric IOLs for lower astigmatism has rarely been investigated [7–9]. In general, the use of toric IOLs is recommended in patients undergoing cataract surgery or refractive lens exchange [10] with regular corneal astigmatism, to be distance vision spectacle free after the surgery. The use of this type of lens during standard phacoemulsification cataract surgery in eyes with pre-existing corneal astigmatism is widely used worldwide by cataract surgeons to enhance visual acuity after implantation.

The hydrophobic acrylic toric IOL (PODEYE TORIC, POD T 49P, Beaver-Visitec International Inc.) with a unique double C-loop design was developed with different cylindrical powers: 1.00, 1.50, 2.25, 3.00, 3.75, 4.50, 5.25, and 6.00 D. It was anticipated that the double C-loop design will prevent IOL rotation caused by capsule contraction. Clinical evaluations in European Union countries have resulted in favorable outcomes showing high rotational stability with good visual acuity and refractive outcomes [11, 12]. However, to the best of our knowledge, the clinical performance of this lens has not been assessed in Asian eyes. The main purpose of this clinical trial is to report the clinical outcomes of the PODEYE TORIC IOL when implanted bilaterally in Japanese patients with cataracts and pre-existing corneal astigmatism.

## Methods

### Participants and study design

This multicentre, single-arm clinical trial was approved by the Investigational Review Boards of Osaka Clinical Trial Hospital (For Chukyo Eye Clinic, Fujita Eye Clinic, and Roppongi Shiba Eye Clinic [Japan]) and Miyata Eyes Hospital (Japan). Written informed consent was obtained from each patient. The study adhered to the tenets of the Declaration of Helsinki and the ‘Ministerial Ordinance on Good Clinical Practice for Medical Devices’ (Ordinance of the Ministry of Health, Labour and Welfare No. 36, 2005). This study was registered at ClinicalTrials.gov (ID: NCT05255029).

This study was designed to assess the outcomes of the PODEYE TORIC IOL (POD T 49P) in groups categorised according to cylinder power: eyes with toric IOLs of cylinder powers of 1.50 D to 6.00 D and 1.00 D cylinders, respectively. Toric IOLs were implanted in the capsules of patients 20 years or older with cataracts from the Japanese population after cataract-removal utilising phacoemulsification and aspiration procedures. The exclusion criteria included ocular pathology influencing postoperative vision, except for cataracts, a history of previous ocular surgery, corneal abnormalities, previous corneal transplant, poor mydriasis, glaucoma, amblyopia, optic nerve atrophy or intraoperative complications.

### Sample size

Primary endpoints of the study included non-inferiority in uncorrected distance visual acuity (UDVA) in the use of PODEYE TORIC with a cylinder power of 1.50 to 6.00 D at 6 months postoperatively and superiority in refractive cylinder values in the use of the 1.00 D cylinder power model. The sample size for each endpoint was calculated as follows: 1) The mean UDVA was estimated as 0.07 logMAR with a 95% confidence interval (CI) of 0.054–0.086 logMAR according to our in-house data. To examine the non-inferiority of UDVA to AcrySof IQ toric IOLs (Alcon) with a standard deviation (SD) of 0.15 [13], 24 eyes or more were required under a margin of 0.10, one-sided significance level of 2.5%, and a power of 90%. Considering a 10% drop-out rate, the required sample size was 29. 2) Similarly, the SD of the refractive cylinder was estimated to be 0.5 D based on our in-house data and previous results [5]. To examine superiority to eyes with non-toric IOL, SN60WF IOL (Alcon) [5] was used with a difference of 0.3 D and SD of 0.5 D, a significance level of 2.5% (one-sided), and a power of 80%. A total of 24 eyes was required. Considering a 10% drop-out, the required sample size was 27. Then, based on sample size calculations, the total sample size was 56.

### IOL and surgery

**Fig. 1:**
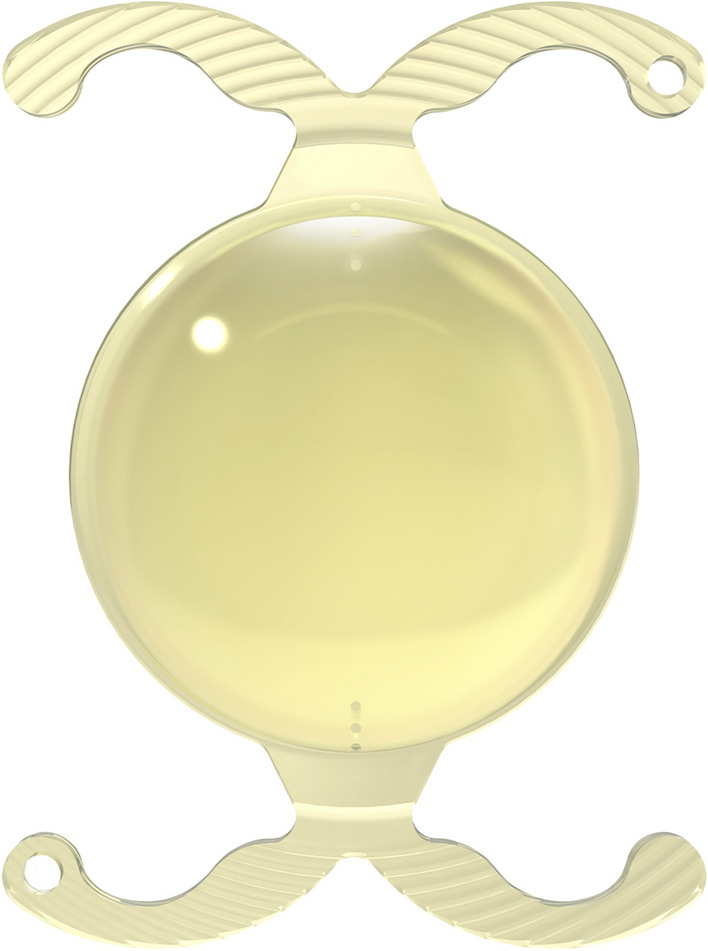
PODEYE TORIC (POD T 49P) intraocular lens with double C-loop haptics (picture property of BVI)

The implanted toric IOLs were the PODEYE TORIC model, and the material was a cross-linked, acrylate/methacrylate copolymer with a blue light absorber (refractive index of 1.53), with a biconvex, aspheric, optic diameter of 6.0 mm and an overall length of 11.4 mm. The IOL possessed haptics of a double C-loop design (Fig. 1), which symmetrically sustained the IOL with four haptics to ensure stability in the capsule. As toric IOLs, seven models with cylinder powers of 1.00, 1.50, 2.25, 3.00, 3.75, 4.50, 5.25, and 6.00D added to the posterior optics were available. The unique haptics consist of four C-loops for better centralisation.

Corneal astigmatism and axial length were measured preoperatively using keratometry and biometry, respectively. Biometry included swept-source optical coherence biometers; IOL Master 700 (Carl Zeiss Meditec AG) in 2 sites, ARGOS (Alcon) in a site, and OA-2000 (Tomey) in one site. Powers of IOLs were determined for emmetropia using the Barrett Universal II formula with the lens constant of 2.15. The toric model and axis position were determined using the Toric Calculator (available at http://www.physioltoric.eu), which is based on the Abulafia–Koch regression formula [14]. Surgically-induced astigmatism at each site was used. After cataract removal through incisions of 2.2 to 3.2 mm, the IOL was implanted in the capsule using a specific injector (Accuject, Medicel AG). The toric IOL axis was aligned at the calculated position using the surgical guidance system at each site. All the participants received standard regimens of preoperative, operative, and postoperative medication.

### Examinations

UDVA, corrected distance visual acuity (CDVA), and manifest refraction (sphere and cylinder) were recorded at 6 months postoperatively. UDVA and CDVA were measured using Landolt ring charts at a 5-m distance under photopic illumination (85–110 cd/m^2^) and converted to logMAR notation for analysis. During CDVA examinations, manifest refractions were recorded, and spherical equivalent (SE) values were calculated.

As misalignment of the toric IOL from the calculated position resulted in the degradation of the astigmatic correction, the IOL axis mark positions were measured 1 day and 3 months after surgery. After mydriasis, an anterior segment image of the implanted IOL was captured and the axis mark position was measured using Photoshop (Adobe).

### Statistical analysis

The primary endpoints were non-inferiority in the UDVA and superiority in the refractive cylinder at 6 months postoperatively. 1) The non-inferiority in UDVA was examined using a toric IOL with a cylinder power of 1.50 to 6.00 D. Because bilateral implantations were included, mixed-effect models for repeated measures (MMRM) were used to calculate the least-squared mean and 95% CI at 6 months postoperatively. The non-inferiority of the AcrySof IQ toric IOL was examined if there was an upper limit of 95% CI <0.2 logMAR (estimated mean of 0.1 logMAR [13] and a margin of 0.1 logMAR). 2) The superiority of the refractive astigmatism in the use of a 1.00 D-cylinder toric IOL over a non-toric IOL was explored. Using MMRM, the least-squared mean and 95% CI of the refractive astigmatism were calculated. The superiority in which the upper limit of 95% CI should be <0.8 D (mean + SD) was examined. The absolute difference between the measured axis mark positions at different times post-surgery was calculated as the IOL rotation, and a median and ratio of <10° were obtained. P<0.025 and P <0.05 were considered as statistically significant for one-sided and two-sided analyses, respectively.

## Results

A total of 64 eyes from 46 patients were included in this study. One case did not satisfy the inclusion criteria and 5 eyes withdrew prior to surgery. Consequently, 58 eyes of 42 patients (20 men and 22 women) completed IOL implantation and were followed-up for 6 months. There were no intraoperative or postoperative complications relevant to toric IOL implantation that influenced postoperative vision, except for 2 eyes with posterior capsule opacification following YAG laser posterior capsulotomy. In 27 eyes, the toric model of the 1.00 D-cylinder was implanted. In 31 eyes, models with a cylinder power of 1.50 to 6.00 D were implanted, consisting of cylinders of 1.50 D (12 eyes), 2.25 D (5 eyes), 3.00 D (4 eyes), 3.75 D (4 eyes), 4.50 D (2 eyes), 5.25 D (2 eyes), and 6.00 D (2 eyes). Table 1 lists the demographic data of all eyes, eyes with a 1.00 D-cylinder toric IOLs, and eyes with 1.50–6.00 D-cylinder models. The mean age of the 1.00 D-cylinder group was lower (P=0.049, t-test) than that of the 1.50–6.00 D-cylinder group, while no differences in the axial length and IOL power were observed (P>0.092).

**Table 1:** Demographic data of the participants.

Toric model	All	1.00 D-cylinder	1.50–6.00 D-cylinder	*P*-value
N, eye/patient	58/42	27/22	31/21	
Age, year	72.1 (7.1) [57, 91]	70.1 (5.7) [59, 80]	74.2 (7.8) [57, 91]	0.049^#^
Axial length, mm	23.7 (0.8) [22.1, 25.6]	23.5 (0.9) [22.1, 25.6]	23.8 (0.7) [22.2, 25.2]	0.29^#^
Corneal astigmatism, D	1.34 (0.94) [0.4, 4.1]	0.73 (0.22) [0.4, 1.1]	1.87 (1.01) [0.7, 4.1]	< 0.001^#^
IOL power, D	20.72 (2.63) [13.5, 26.0]	21.35 (2.67) [14.5, 26.0]	20.18 (2.52) [13.5, 24.5]	0.092^#^
CDVA, logMAR	0.23 (0.31) [-0.08, 2.00]	0.18 (0.16) [-0.08, 0.70]	0.27 (0.40) [-0.08, 2.00]	0.81*
MRSE, D	0.06 (2.09) [-5.00, 3.75]	0.24 (1.89) [-4.00, 3.75]	-0.10 (2.27) [-5.00, 3.38]	0.54^#^

Mean (SD) [range]

^#^: unpaired t-test between 1.00 D-cylinder and 1.50–6.00 D-cylinder groups. *: Mann-Whitney U-test between 1.00 D-cylinder and 1.50–6.00 D-cylinder groups

IOL, intraocular lens; CDVA, corrected distance visual acuity; logMAR, logarithm of the minimum angle of resolution; MRSE, manifest refraction spherical equivalent; D, diopter

**Fig. 2:**
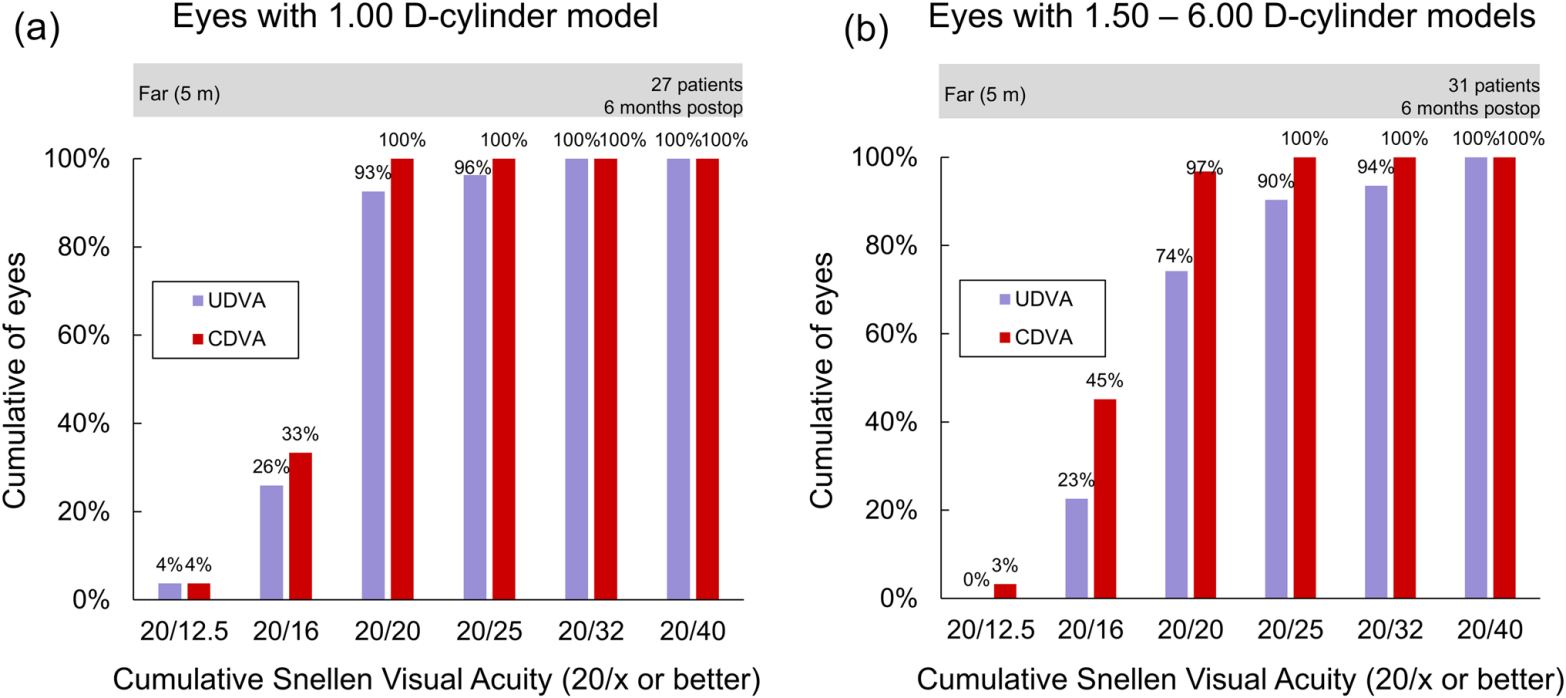
Cumulative percentage of eyes attaining uncorrected and corrected distance visual acuities (UDVA and CDVA, respectively) with the 1.00 D-cylinder (a) and 1.50–6.00 D-cylinder (b) models at 6 months postoperatively

**Fig. 3:**
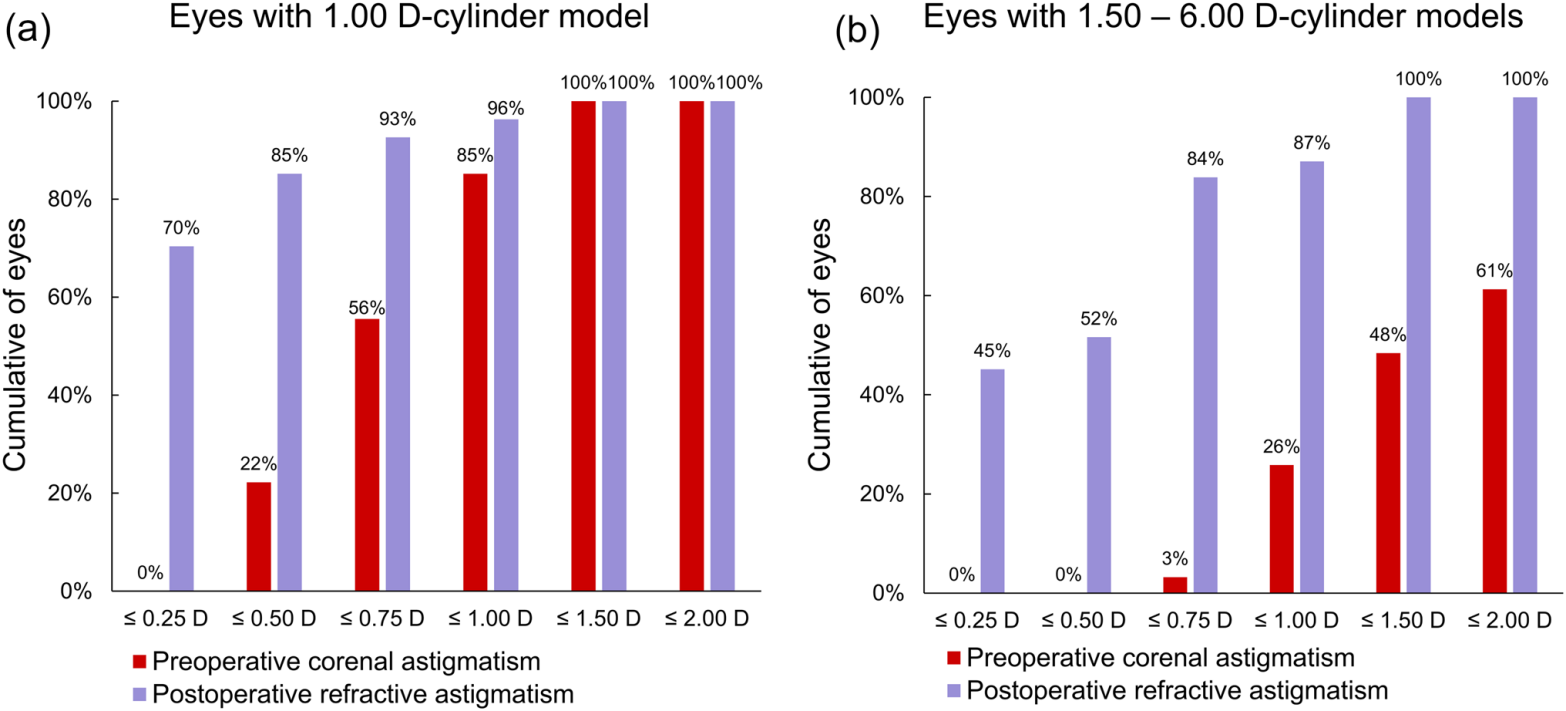
Cumulative histogram of the magnitude of the preoperative corneal and postoperative refractive astigmatism with the 1.00 D-cylinder (a) and 1.50–6.00 D-cylinder (b) models, vertexed to the corneal plane

Table 2 illustrates the postoperative visual acuity and manifest refractive spherical equivalent (MRSE). No differences in the UDVA and CDVA were observed (P >0.26, Mann–Whitney U test). The mean corneal astigmatism was 1.32 ± 0.97 D, which differed by 0.02 D from the pre-operative value (P = 0.74, paired t-test). The mean postoperative refractive prediction error was 0.28 ± SD: 0.33 D. Figure 2A-B illustrates the cumulative percentages of eyes achieving UDVA and CDVA with the 1.00 D-cylinder (a) and 1.50–6.00 D-cylinder (b) models. In the 1.00 D-cylinder model, the UDVA and CDVA of 20/20 or better were obtained in 93% and 100% of eyes, respectively, while they were 74% and 97% respectively in the eyes using 1.50–6.00 D-cylinder models (P =0.064 and 0.35, chi squared test). In the MRSE, no differences in the mean sphere and SE (P >0.26, t-test) between the two types were noted, while the cylinder with 1.00 D-cylinder model was closer to emmetropia (P =0.034, t-test) with a mean difference of 0.11 D. Figure 3A-B demonstrates the cumulative histogram of the magnitude of the preoperative corneal and postoperative refractive astigmatism with the 1.00 D-cylinder (left) and 1.50–6.00 D-cylinder (right) models. Refractive astigmatism was vertexed to the corneal plane [14]. In the 1.00 D cylinder model, postoperative refraction of 0.5 D or less was achieved in 85% of patients, while only 52% was achieved using the 1.50–6.00 D cylinder models for higher preoperative corneal astigmatism (P =0.0066, chi squared test).

**Table 2:** Postoperative uncorrected/corrected visual distance acuities (UDVA/CDVA) and manifest refractions.

Toric model	All (N = 58)	1.00 D-cylinder (N = 27)	1.50–6.00 D-cylinder (N = 31)	*P*-value
UDVA, logMAR	$-$ 0.05 (0.10) [$-$ 0.30, 0.22]	$-$ 0.07 (0.10) [$-$ 0.30, 0.15]	$-$ 0.04 (0.11) [$-$ 0.18, 0.22]	0.26*
CDVA, logMAR	$-$ 0.11 (0.07) [$-$ 0.30, 0.10]	$-$ 0.10 (0.07) [$-$ 0.30, 0.00]	$-$ 0.12 (0.08) [$-$ 0.30, 0.10]	0.38*
Manifest refractions, D Sphere Cylinder SE	0.22 (0.37) [$-$ 0.25, 1.25] $-$ 0.30 (0.35) [$-$ 1.25, 0.00] 0.08 (0.30) [$-$ 0.75, 0.75]	0.17 (0.29) [0.00, 1.00] $-$ 0.19 (0.28) [$-$ 1.00, 0.00] 0.07 (0.23) [$-$ 0.25, 0.63]	0.27 (0.43) [$-$ 0.25, 1.25] $-$ 0.39 (0.39) [$-$ 1.25, 0.00] 0.08 (0.36) [$-$ 0.75, 0.75]	0.26^#^ 0.034^#^ 0.89^#^

Mean (SD) [range]

^#^: unpaired t-test between 1.00 D-cylinder and 1.50–6.00 D-cylinder groups. *: Mann-Whitney U-test between 1.00 D-cylinder and 1.50–6.00 D-cylinder groups

logMAR: logarithm of minimum angle of resolution; D: diopters; SE: spherical equivalent

Non-inferiority in UDVA and superiority in refractive astigmatism were examined. 1) The least-squared mean UDVA of 31 eyes with IOLs with 1.50 to 6.00 D cylinders was $-$ 0.030, with two-sided 95% CI of $-$ 0.072 and 0.012. The upper limit (0.0012 logMAR) was < 0.20 logMAR to ensure non-inferiority to the control lens [12]. 2) The least squared mean of 27 eyes with 1.00 D-cylinder IOLs was 0.156 D with a two-sided 95%CI of 0.057–0.254 D. The upper limit was 0.254, which was lower than the 0.8 D astigmatism, and the superiority to the non-toric lens [5] was verified.

In relation to IOL rotation (n=57), we found a mean absolute rotation value of 2.70 ± 2.88° (ranging from 0 to 16°). 56 eyes (98.25%) had a rotation <10°, and, specifically, we found 45 eyes (78.95%) with a rotation less than 5°, 11 eyes (19.30%) between 5 and 10° and 1 eye (1.75%) between 10 and 20°.

## Discussion

In the review and meta-analysis [15], the pooled mean absolute rotations of the SN60TT IOL and SN6ATT IOLs (Alcon) were 2.94° (95% CI, 1.72-4.16) and 2.55° (95% CI, 1.70-3.40), respectively. Our results reveal a mean absolute rotation value of 2.70 ± 2.88°, comparable to the previous ones. The ISO11979-7:2018 indicates that the absolute value of rotation should be less than 10° in 90% of eyes implanted after surgery [16]. In our cohort, the outcomes obtained broadly fulfil this criterion, since our percentage for this value was 98.25%. One of the specific characteristics of this IOL is the excellent manoeuvrability, as the IOL can be rotated in both directions (clockwise and anticlockwise), allowing the alignment of the toric IOL with the desired axis during surgery.

It is well known that a misalignment of less than 10° can lead to a minimal to moderate loss of cylindrical correction, being severe for higher values with the loss of the whole cylindrical correction [17, 18]. The postoperative UDVA of eyes with PODEYE TORIC IOL with a cylinder power of 1.50 to 6.00 D and refractive astigmatism of eyes with the 1.00 D-cylinder model at 6 months indicated the non-inferiority to AcrySof IQ toric IOL and the superiority to non-toric IOL, respectively, in the use for Japanese patients. Table 3 compares the current results with previous evaluations with the PODEYE TORIC in different countries [11, 12], which included models with cylinders of 1.00 D until 6.00 D. The current results are similar to the previous outcomes.

**Table 3:** Comparison with previous evaluations.

	Chassani C et al. [11] N = 107 in EU	Ang RET et al. [12] N = 94 in Asia and EU	Current results N = 58 in Japan
Design/ observation period	Retrospective/ 6 months	Prospective/ 4–6 months	Prospective/ 6 months
UDVA (only emmetropia targeted)			
Mean, logMAR	$-$ 0.01 (SD: 0.13)	0.08 (SD: 0.10)	$-$ 0.05 (0.10)
% of 20/20 or better	78.0%,	33.0%	82.8%
Refractive cylinder			
Mean, D	0.37 (SD: 0.29)	0.36 (SD: 0.35)	$-$ 0.30 (0.35)
% of ≤0.50 D	84.1%	76.5%	67.2%
IOL rotation			
Mean	2.07 ° (SD: 2.17)	1.22 ° (SD: 2.21)	2.70 ° (SD: 2.88)
% of <10 °	98.9%	97.8%	98.25%

UDVA, uncorrected distance visual acuity; logMAR, logarithm of the minimum angle of resolution; D, diopters; IOL, intraocular lens.

A recent systematic review and single-arm meta-analysis show that the pooled mean absolute postoperative rotation of all toric IOLs was 2.36° (95% CI, 2.08-2.64) [15], while the postoperative rotation found in eyes implanted with toric IOLs depends on many aspects of the IOL material and its design. Double-loop haptics and plate loop demonstrate even greater rotational stability than standard loop haptics, due to the increased number of contact points between the haptics and the capsular bag (increasing the total friction). Note that hydrophobic acrylic IOLs exhibit stronger adhesion owing to the charge effect and higher fibronectin content [19] and it is indicated that toric IOLs made with hydrophobic acrylates exhibit superior postoperative rotational stability compared to those made with hydrophilic acrylates [20, 21].

The overall length of the IOL (11.4 mm) is shorter than conventional IOLs with standard C-loops (typically, 13 mm). Evaluation of postoperative capsule bag diameters (CBD) of 70 eyes by using implanted open capsular tension rings resulted in the range of 9.82 to 10.88 mm [22]. Another measurement of 24 eyes using ultrasound biomicroscope indicate that the CBDs were below 11.15 mm [23]. These evaluations show that the overall length of 11.4 mm is sufficient for the contacts to the normal capsule bag. In the evaluation of mechanical stability of current IOLs including POD F GF of 11.4-mm overall length and double-C loops and SN6CWS (Alcon) with 13,0-mm overall length and C-loops, all types of IOLs confirmed mechanical contact up to the diameter of 11.0 mm [24]. These findings demonstrate that the PODEYE TORIC IOL allows contact to the capsule bag, whenever the CBD is not singularly large.

The good refractive outcomes in our sample allowed us to obtain good visual acuity, since residual uncorrected values of sphere and cylinder degrade visual acuity. With the use of swept-source optical coherence biometers and the Barrett Universal II formula, postoperative refractive prediction error (mean: 0.28 D) was sufficiently small. Figure 2A-B shows the cumulative percentages of eyes achieving UDVA and CDVA for the two groups assessed. In the 1.00 D-cylinder model, the UDVA and CDVA of 20/20 or better were obtained in 93% and 100% of eyes, respectively, while there were 74% and 97% in the eyes with the use of 1.50–6.00 D-cylinder models, respectively. These outcomes are better than those reported by Chassain et al. [11] in their cohort, who found 96.3% of eyes with a cumulative monocular CDVA of 20/20, and for Ang et al. [12] with 58.5% of eyes with a monocular cumulative CDVA of 20/20.

Clinical outcomes in the use of toric IOLs with a cylinder of 1.00 D were comparable with the use of other models. In a recent comparison between toric IOLs with a 1.0 D cylinder and non-toric IOLs, the improvement in the use of toric IOLs was limited, with a minimal reduction in postoperative refraction and improvement in UDVA [8]. Holladay addressed the importance of toric IOL correction as there are factors influencing postoperative refractive astigmatism such as IOL tilt, decentration, and posterior corneal astigmatism [25]. With such minor factors, visual function is degraded when presbyopia-correcting IOLs are used. Orts-Vila et al. evaluated the outcome of the hydrophilic trifocal IOL POD FT (the same haptic design as the monofocal toric IOL analysed in the present study) with a 1.00 D cylinder in 26 eyes of 22 patients [26]. The postoperative refractive cylinders were 0.5 D or less in all eyes and 0.25 D or less in 73.1% of eyes, and 81% of eyes achieved a UDVA of 20/20 or better. In the trifocal toric AcrySof IQ PanOptix IOL TFNT20 (Alcon) with a cylinder power of 1.0 D for 41 Japanese eyes, all and 90% of eyes achieved the postoperative cylinders within 0.5 and 0.25 D, respectively, 1 month after surgery [9], resulting in 90% of eyes achieving a UDVA of 20/20 or better. While such a conspicuous effect was not observed with monofocal IOLs, the 1.00 D cylinder model would be effective for correcting low astigmatism.

The study had some limitations. First, this prospective clinical trial was designed with no control group for rigorous evaluation. This clinical trial was designed for IOL approval in Japan utilizing historical records, and toric IOL with 1.0 D cylinder had not been available. It is important to compare these results of toric IOLs with a double C-loop with a conventional toric IOL with C-loops under identical procedures including toric model calculations. Next, the posterior corneal astigmatism was not measured. The influence of against the rule astigmatism on the posterior cornea was included. In the study, the Abulafia–Koch regression formula [14] which compensates with theorical posterior astigmatism was used, however, postoperative astigmatism would vary with the posterior astigmatism of each eye. Third, it was hard to compare outcomes of 1.00 D-cylinder model, as other toric IOLs with 1.0 D cylinder had not been available. Although, the current results demonstrate that toric IOLs are effective for low astigmatism, more precise examination and model calculation are required [25].

In conclusion, the present clinical trial analysing the PODEYE toric IOL implantation in Japanese eyes suggests that this lens provides excellent refractive and visual outcomes in patients with pre-existing corneal astigmatism undergoing cataract surgery. The PODEYE toric IOL with the double C-loop design allows good rotational stability, minimising the impact of lens rotation and therefore providing accurate correction of astigmatism.
